# Identifying Ortholog Selective Fragment Molecules for Bacterial Glutaredoxins by NMR and Affinity Enhancement by Modification with an Acrylamide Warhead

**DOI:** 10.3390/molecules25010147

**Published:** 2019-12-30

**Authors:** Ram B. Khattri, Daniel L. Morris, Stephanie M. Bilinovich, Erendra Manandhar, Kahlilah R. Napper, Jacob W. Sweet, David A. Modarelli, Thomas C. Leeper

**Affiliations:** 1 Department of Physiology and Functional genomics, University of Florida, Gainesville, FL 32610, USA; rbk11@ufl.edu; 2 Department of Chemistry and Biochemistry, The University of Akron, Akron, OH 44325, USA; danmorris4127@gmail.com (D.L.M.); krn14@zips.uakron.edu (K.R.N.); jws81@zips.uakron.edu (J.W.S.); dmodarelli@uakron.edu (D.A.M.); 3 Department of Pediatrics and Human Development, Michigan State University, East Lansing, MI 48824, USA; bilinovi@msu.edu; 4 Department of Chemistry, Berea College, Berea, KY 40404, USA; emanandhar@gmail.com; 5 Department of Chemistry and Biochemistry, Kennesaw State University, GA 30144, USA

**Keywords:** glutaredoxin, FBDD, STD, HSQC, trNOE, acrylamide warhead, docking, covalent inhibition

## Abstract

Illustrated here is the development of a new class of antibiotic lead molecules targeted at *Pseudomonas aeruginosa* glutaredoxin (PaGRX). This lead was produced to (a) circumvent efflux-mediated resistance mechanisms via covalent inhibition while (b) taking advantage of species selectivity to target a fundamental metabolic pathway. This work involved four components: a novel workflow for generating protein specific fragment hits via independent nuclear magnetic resonance (NMR) measurements, NMR-based modeling of the target protein structure, NMR guided docking of hits, and synthetic modification of the fragment hit with a vinyl cysteine trap moiety, i.e., acrylamide warhead, to generate the chimeric lead. Reactivity of the top warhead-fragment lead suggests that the ortholog selectivity observed for a fragment hit can translate into a substantial kinetic advantage in the mature warhead lead, which bodes well for future work to identify potent, species specific drug molecules targeted against proteins heretofore deemed undruggable.

## 1. Introduction

Described herein is the development of a novel type of covalent inhibitor with selective reaction kinetics for the *P. aeruginosa* glutaredoxin (PaGRX). This work was inspired by the need to develop new classes of drug molecules directed against unconventional drug targets using mechanisms that may circumvent common bacterial antibiotic avoidance strategies. Covalent inhibitors are re-emerging as tools for antineoplastic therapies [1], but there are compounds that have seen widespread use besides the serine β-lactamase inhibitors [2]; perhaps because of their obvious pharmacokinetic limitations. However, considering the rapid evolution of bacterial resistance to conventional drug compounds, investigation of all therapeutic strategies is required to prevent resistance to or at least extend the lifetime of usefulness of antimicrobial agents. In *P. aeruginosa* resistance is often one of two routes: (1) overexpressing efflux pathways, or (2) mutations within target proteins that render a drug ineffective [3]. The present strategy is to combine a covalently reactive functional group, eliminating the efflux problem, with targeting moieties selective for species specific proteins from biochemical pathways that would have been avoided due to cross-reactivity risks for the host organism. These atypical protein targets are less likely to contain existing mutations that convey resistance and thus may avoid resistance longer. In addition, infectious colonies are less likely to generate a viable mutation if the targeted pathway is central to the bacterial metabolism. Our results show that comparative screening of drug precursors interacting with bacterial proteins and the related human homolog may enable identification of base scaffolds selective for the bacterial protein that can be used to generate covalent inhibitors that avoid cross-reactivity in vivo.

Orthologous proteins are those that share their gene sequence origins in a common evolutionary ancestor. They are usually highly homologous, structurally similar, and perform the same functions, but contain subtle variances in sequence due to natural evolutionary divergence. Of particular interest are differences proximal to conserved enzyme active sites, since the actual active sites are expected to be invariant [4]. Ligands that have special affinity for these nearby binding sites can be optimized for inhibition by chemical elaboration into the active site. A popular option for ligand growing is the use of an electrophilic functional group (warhead) that can form a covalent bond with a nucleophilic residue on the target protein. When attached at a suitable position on the lead compound (driving group), the warhead is directed toward the nucleophilic side chain and a covalent binding event can occur; usually driven by the Michael addition reaction [5]. With the lead compound now serving as a driving group, it can increase residence time of the warhead near the active site. This confers a kinetic advantage over the warhead as a free ligand [6]. The driving group also enables target specificity, eliminating some of the concerns associated with covalent inhibitors [7]. A comparison of small ligand fragments interacting uniquely with orthologous proteins is the foundation for our approach to discovering species selective lead compounds. Presently, we focus on a set of glutaredoxin proteins (GRXs) as a model system to describe a novel, species selective fragment-based drug discovery (FBDD) strategy.

FBDD is an established method for the discovery of lead molecules that can be developed into higher affinity drug-like compounds for protein targets. This screening method uses libraries of fragment molecules (1 × 10^4^ compounds weighing between 150–300 Daltons) to probe the surfaces of proteins for small, previously unknown binding epitopes. FBDD methods include highly sensitive techniques such as NMR [8], X-ray crystallography [9], surface plasmon resonance [10], weak affinity chromatography [11], native mass spectroscopy [12], and calorimetry [13], among others. FBDD is often considered to be more effective than high throughput screens (HTS) for searching chemical combinatorial space and may produce fewer false negatives than with larger molecules [14,15,16,17] and when combined with structure-based methods can greatly accelerate the pace of drug discovery [18]. 

While there are several options available for NMR-based FBDD [19], we chose to use a modified saturation transfer difference (STD) NMR-based screening strategy against three orthologous GRXs [20]. In this method ^1^H-NMR spectra are collected in a pair of experiments where in one a pulse is introduced on the methyl frequency (on-resonance) to irradiate the protein target, completely saturating all protein ^1^H signals via spin diffusion. In the second experiment, the saturation pulse is applied off-resonance and does not saturate the protein at all. In the former experiment, saturation is transferred through space to the ligand if it is interacting with the protein in some way. Ligand dissociation after saturation transfer results in a partial loss of signal intensity for the ligand resonances as the saturated ligand population contributes to the overall signal from the free ligand pool [21]. What is distinctive about our screen is that we are performing it on three different but evolutionarily related protein orthologs using the exact same conditions and panel of fragments to identify sets of common binders, non-binders, and ortholog preferential binders.

**Figure 1:**
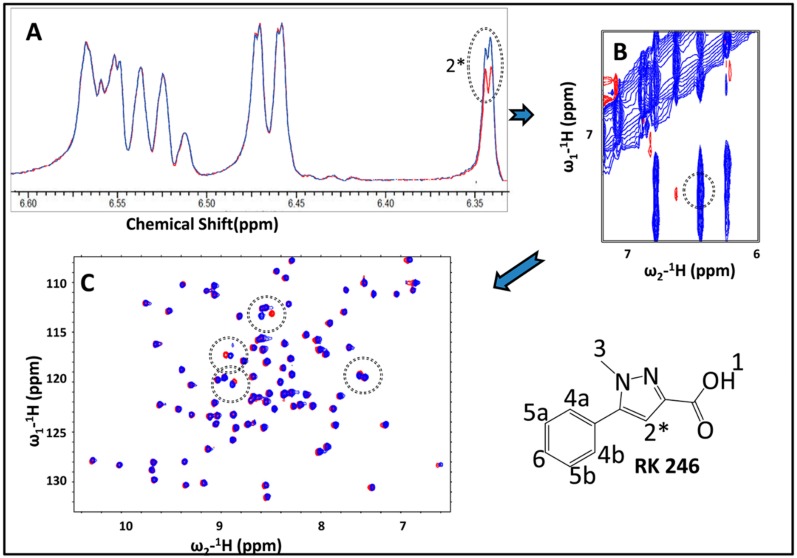
1D and 2D NMR experiments for RK246 against BrmGRX. (**A**) STD NMR spectrum for a fragment mixture, (red: On-resonance, blue: Off-resonance), (**B**) trNOE NMR spectrum for a fragment mixture, (red: Negative contour, blue: Positive contour), (**C**) ^15^N HSQC NMR spectrum for 5 mM of RK246 against 0.25 mM of reduced BrmGRX (red: Apo, blue: With fragment).

To verify positive hits obtained from the STD screen, we employed transfer nuclear Overhauser effect (trNOE) and ^15^N heteronuclear single quantum coherence (HSQC) chemical shift perturbation (CSP) as complementary forms of cross-validation for each fragment [22,23]. In trNOE, when a fragment binds transiently to a large molecule it inverts the sign of the NOE cross-peaks. This change in sign is due to change in molecular tumbling rate of the ligand as it moves in congruence with the target and confirms that it is a binder of that protein [19,24]. ^15^N HSQC CSP screening identifies residue specific changes in the chemical environment of the protein target when bound to the ligand. This approach observes spectral perturbations (i.e., CSPs) to characterize a binding event with a ligand from the protein’s perspective [25]. As each peak in the HSQC corresponds with a unique residue on the protein, perturbations exceeding the standard deviation can be said to correlate with residues that interact with the ligand [26]. Figure 1 depicts these three methods used in congress to identify an exemplary fragment, RK246.

After screening and validation of hits, the next important step in any drug design campaign is lead optimization [27]. A good hit in FBDD is not only determined by its binding affinity with the target macromolecule, but also by its ligand efficiency (LE), which is essentially affinity normalized for size. There are several effective drug candidates that are known to show dissociation constants in the nanomolar range but have LE of only 0.3 $\frac{\mathrm{kcal}}{\mathrm{mol}\;\times \;n}$ (*n* = heavy atoms) or larger [28]. Hits with higher LE values are believed to be more useful as leads and are often good candidates for chemical elaboration or optimization. This can involve linking or merging weak fragments that probe adjacent binding epitopes on a target molecule for large payoffs in affinity [29]. In the present study, growing the lead into the thiolate-containing active site afforded consideration of a chemically reactive cysteine trap moiety that should allow covalent inhibition of the protein target.

GRXs are characterized by a dithiol containing active site with a Cys-X-X-Cys motif at the N-terminal end of their enzymatically active helix [30]. Using one or both active site Cys thiolates, GRXs can catalyze the reversible reduction of a range of disulfide containing molecules. By taking advantage of the nucleophilic properties of these active site thiolates, it is possible to develop inhibitors for GRXs. Thiols and thiolates are prime targets for chemical modification, where the right compound could produce covalent inhibition of a target protein’s enzymatically important cysteine residues. Indeed, covalent alkylation of enzymatically important cysteines have already been explored to produce irreversible inhibitors of epithelial growth factor receptors (EGFR) [5,31,32], sortase [33], and some receptor tyrosine kinases [34]. Cys alkylation is achieved by bringing a vinyl containing functional group into close proximity with the active site thiol(ate). Once in place, a covalent bond is formed between protein and ligand via a Michael addition reaction, where the Michael donor is the thiol or thiolate sulfur anion of a target cysteine and the Michael acceptor is the vinyl group [35]. In the case of EGFR inhibitors, acrylamide-based functional groups were chemically synthesized into existing drug compounds creating vinyl containing warhead groups and increasing potency. Such functional groups are termed acrylamide warheads [5].

Major concerns associated with warhead-based inhibitors are toxicity, cellular efficacy, and off-target reactivity. In early drug design campaigns researchers tried to avoid smaller fragments containing reactive electrophilic groups. These were considered to be promiscuous hits that can react with any target protein and are very difficult to optimize into selective leads [34]. More recently covalent inhibitors have been regaining researchers’ interest as a final optimization step in designing therapeutic compounds [36]. Warheads based on highly selective compounds can maintain the parent molecule’s specificity and potency for a particular target protein, setting the stage for further optimization into drug-like compounds with the potential for regulatory approval [37].

Compounds containing acrylamide warheads share two basic characteristics: (1) an electrophile reactive moiety, i.e., the warhead, and (2) a driving group. The driving group, typically a heterocyclic molecule, forms the bulk of the compound and provides target specificity and affinity. This driving group is also responsible for positioning the reactive warhead into the active site. The attached acryl electrophilic warhead should have reasonable fit in the active site, low off-target reactivity, and have its reactive centers properly oriented to interact with a target nucleophile [38]. Together these produce highly selective, irreversible inhibitors. While these types of covalent inhibitors have been met with resistance from the medicinal chemistry community in the past, it is becoming a necessity to explore every option to create new antibiotics in the war against bacterial resistance. Covalent inhibitors side-step many of the conventional mechanisms that bacteria use to deal with damaging molecules. Adding species selective elements to covalent inhibitors also affords the advantage of targeting proteins involved in critical metabolic pathways, rendering resistance by mutation a less successful option for bacteria. In the present study, we describe the interaction of a novel acrylamide warhead ligand derived from a fragment driving group with PaGRX. The fragment lead identified as PaGRX selective via the three NMR methods was chemically linked with an acrylic acid moiety to form a more potent lead molecule as an acrylamide warhead. PaGRX selectivity was maintained even after fusion to a chemically reactive warhead and a significant increase in warhead reaction rate was monitored via kinetic NMR experiments.

## 2. Results and Discussion

### 2.1. Identifying Orthologous GRX Candidates

**Figure 2:**
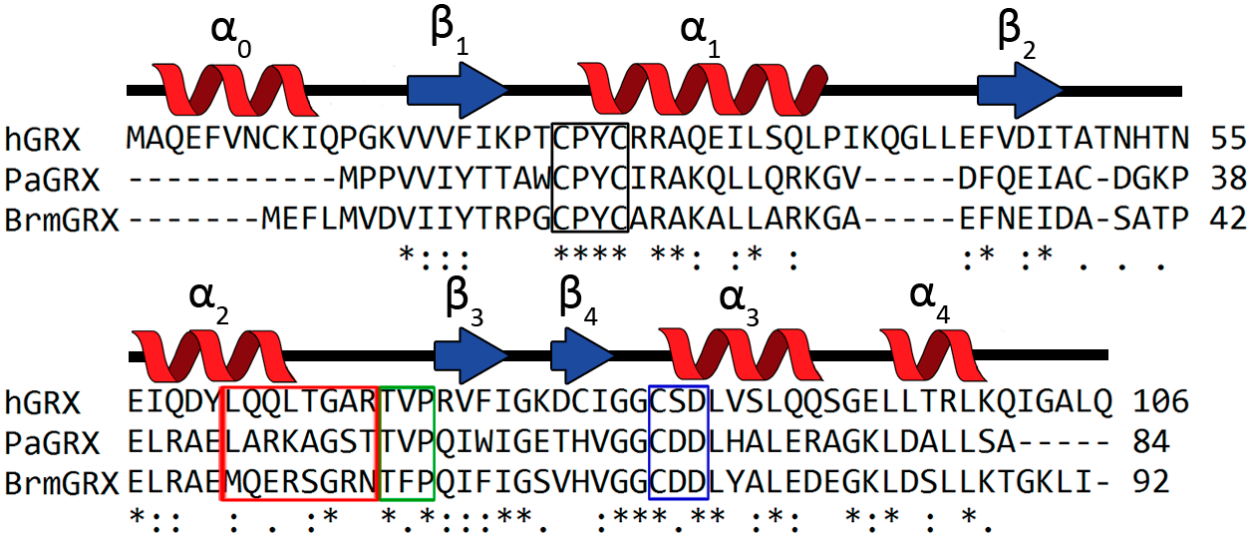
Multiple sequence alignment of the bacterial GRXs from Brucella melitensis (BrmGRX) and Pseudomonas aeruginosa (PaGRX) compared with human GRX1 (hGRX) using Clustal Omega (EMBL-EBI). Less than 40% sequence similarity is observed between the human and bacterial proteins. Red, green, and blue boxes highlight the species specific helix, Thr-Val-Pro (TVP), and Cys-Asp-Asp CDD motifs respectively. The black box highlights the conserved CPYC active site. An asterisk indicates positions with a fully conserved residue, colons indicate conservation with strongly similar properties, and periods indicate conversation with weakly similar properties.

It can be quite challenging to identify orthologous proteins between eukaryotes and bacteria because of extreme sequence divergence between the two domains, the presence of multiple paralogs in one or both species, and a lack of conserved genomic architecture between the organisms of interest. However, sequence comparisons can provide an indication of which homologs are most similar and hence most likely to be orthologs. Sequence alignment using blastp [39] of human glutaredoxins 1–5 with PaGRX reveals that the most conserved sequence is hGRX1, with 37% identity and 66% similarity. The second most conserved was hGRX2 with 33% identity and 52% similarity, but it has a different active site sequence: Cys-Ser-Tyr-Cys (CSYC) in hGRX2 versus Cys-Pro-Tyr-Cys (CPYC) for hGRX1 and the bacterial GRXs. Besides the active site variance, hGRX2 is also known to use GSH to form iron sulfur clusters rather than using cytoplasmic glutathione (GSH) for reversible disulfide reduction and so would be expected to have a non-homologous biochemical role. For comparison, PaGRX and *Brucella melitensis* GRX (BrmGRX) have 61% identity and 70% similarity over 82 residues. Comparing BrmGRX to human glutaredoxins also revealed hGRX1 as the most likely ortholog with 38% identity and 65% similarity while hGRX2 has only 27% identity and 47% similarity to the BrmGRX protein. Structural comparison of hGRX1 to *E. coli* GRX and BrmGRX indicated similarities in the overall fold and structure of the proteins [4]. Thus, we propose that hGRX1 is the human glutaredoxin paralog that is the most closely related functional and structural ortholog to the bacterial sequences studied here. There are several regions of interest on these GRXs that can contribute to fragment selectivity within the binding pocket (Figure 2). It is the goal of this work to survey the sequence variance among species here that result in structural explanations for fragment selectivity.

### 2.2. Fragment Screening of the Three Orthologous Proteins

**Figure 3:**
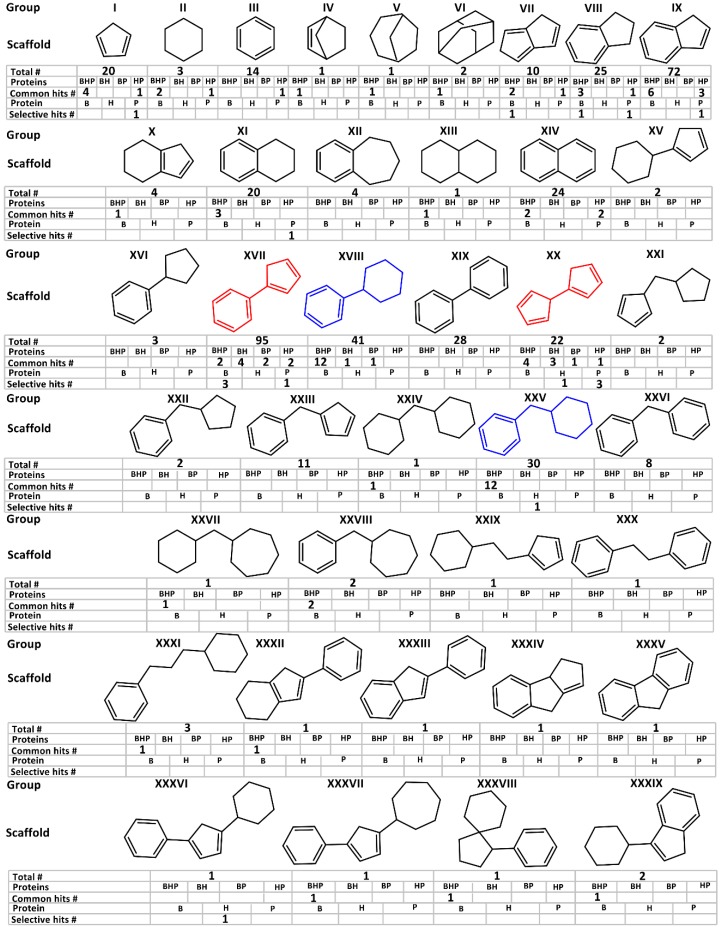
Scaffold groups for the 463 fragment library shown without hetero-atoms and side chains. Total #—Indicates the number of member fragments in each group. Common hits #—Represent number of hits showing significant STD effects for all three GRXs. B stands for BrmGRX, H is hGRX1 and P is PaGRX. Selective hits #—Represent the number of selective hits in each group showing significant STD effects with each unique GRX.

Using ligand observed STD NMR, a library of 463 compounds was screened for binding against the three glutaredoxin orthologs. One key aspect of this screen is that all three GRXs were screened against the same library under similar conditions, thus enabling meaningful cross comparisons of resulting hits. This fragment library is a subset of the Ro3 diversity Maybridge library supplemented with additional commercially available small molecules and consists of 39 dissimilar substructure scaffolds (Figure 3).

**Figure 4:**
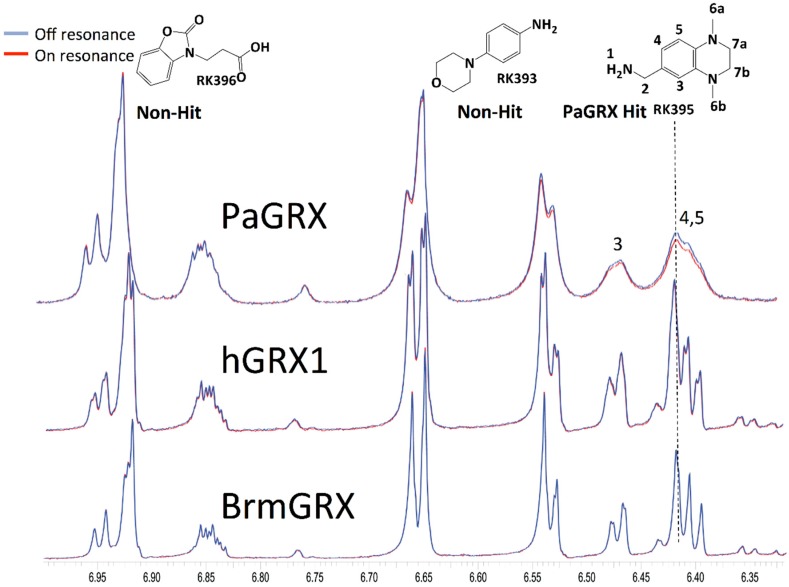
STD spectra of a fragment mixture recorded on the three glutaredoxins with PaGRX, hGRX1, and BrmGRX. Fragment RK395 selectively binding to PaGRX. Some broadening effects are observed for RK395. Blue: Off-resonance spectrum, and red: On-resonance spectrum.

Each fragment was selected with different numbers of heteroatoms and side chains to increase chemical diversity and therefore vary binding ability with proteins. Variance in molecular weight, number of rings, number of heavy atoms, number of hetero-atoms, and log P values for the library are shown in Figure S1 and average values express adherence to the “rule of three” principles [40]. Fragments were initially screened in mixtures of five to seven compounds and hits exceeding the STD threshold values within mixtures were followed up both for reproduction of the STD magnitude and as part of cross-validation with 2D trNOE. An example hit, RK395, with STD-observed selectivity for PaGRX is shown in Figure 4. In later experiments this fragment would also induce resonance broadening in the protein-observed HSQC, indicative of intermediate exchange on the chemical shift time scale and hence tighter binding than a typical, fast exchanging fragment.

**Figure 5:**
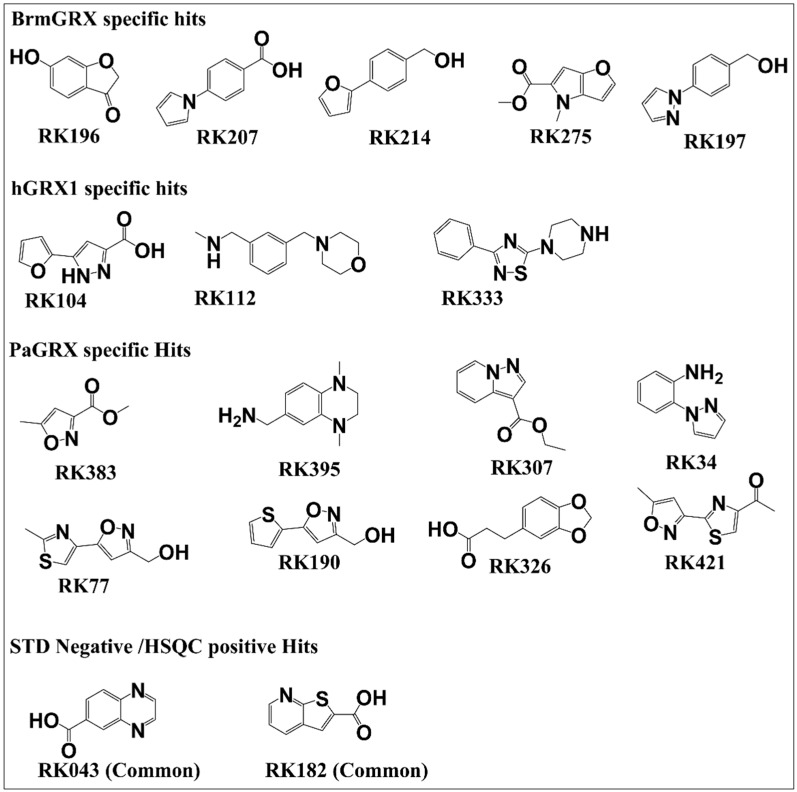
Structures for specific hits and STD NMR negative hits.

A library wide hit rate of ~23% was obtained for the three proteins, which is consistent with hit rates from other STD screens with similarly sized libraries [41,42,43]. The selective hit rate for each individual protein dropped to 1–2%. The overall hit rate is expected to be much larger than selective hit rate because many fragment libraries contain non-selective binders referred to as Pan Assay INterference Substances (PAINS) [44]. From the overall hit rate of 23% (107 out of 463 fragments), 66 fragments were positive hits for all three orthologous proteins and are considered to be *common hits*; note that many but not necessarily all of these *common hits* are expected to be PAINS. Common hits that are not generic protein-binding PAINS are of great interest to us for designing broad-spectrum inhibitors of GRXs as part of future studies. Of the remaining hits, most bind to two of the three proteins and are considered *shared hits*. Finally, any hit that exceeded the STD threshold for only one of the three proteins, the most restrictive category, was deemed a *selective hit*. Examples of selective hits are shown with structures in Figure 5. Among these hits, selective and shared hits for BrmGRX and PaGRX are of great interest for antibiotic development while hGRX selective hits are prospective antitumor compounds.

Analysis of the average physicochemical properties of common, shared, and selective hits revealed similar patterns across classes of binding properties (Figure S2). For all parameters, the variations found were about 13% or smaller. Average logP values are higher for common hits, indicating that these are slightly more hydrophobic than the whole library. The largest difference was observed for average molecular weight of common hits as compared to the library; potentially due to the presence of large alkyl groups conferring hydrophobicity. Similar results were reported by other fragment screening projects [45,46]. However, for selective hits to BrmGRX and hGRX1, average logP values are slightly lower than the library suggesting that the prevalence of particular functional groups rather than non-specific hydrophobic interactions may be important for this selectivity. For BrmGRX selective hits, the average logP value was found to be 22% (1.18) lower than for the library and for hGRX1, it is 40% (1.0) lower. 

### 2.3. Hit Validation with trNOE and ^15^N HSQC

Selective fragment affinities were qualitatively validated using trNOE. More than 50% of positive hits determined by STD showed positive results in trNOE as well and were validated to bind with their respective proteins. Reduction in the number of hits might be because of inherent differences in sensitivity to binding between the two techniques. STD NMR is comparatively more sensitive to the detection of weak interactions between fragments and target proteins as long mixing times can cause signal loss during trNOE [23,47]. Consequently, only half of the STD derived positive hits were found to pass selectivity tests with trNOE, but this validation is an important confirmation of fragment binding and fragment that pass both STD and trNOE filters are more promising lead candidates. Compounds RK144, RK196, RK207, RK214, RK246, RK275, RK307, RK326, and RK395 were determined to selectively interact with one or both of the bacterial GRXs, but not with hGRX1.

2D ^15^N HSQC NMR experiments using isotopically labeled GRXs were used to verify selective fragment binding from the protein’s perspective. A further subset of hits determined from the previous methods display either chemical shift perturbations (CSPs) or selective resonance broadening, both indications of a binding event occurring from the protein’s perspective. Statistical analysis of the degree of perturbation across each amino acid peak indicates which residues on the target protein are involved in the binding event. Mapping these perturbations to the protein structure identifies which binding site the lead fragment is occupying and the side chains of these residues can be used as restraints in docking simulations; as described below, cross-comparison of these CSP locations with other computational methods (FTMap etc.) can provide anchor points useful for restrained docking studies. Possible explanations for the reduced CSP for certain fragments could be due to the absence of strong ring current effects independent of binding strength which is why we tend to use STD and trNOE first and HSQC as a validation tool rather than a primary screen [23,48,49,50].

### 2.4. Dissociation Constant and Ligand Efficiency 

A small subset of hits that had both moderate selectivity as determined by STD/trNOE NMR and obvious CSPs in ^15^N HSQC NMR titration experiments were the focus of further analysis. These were considered the *best hits*. Dissociation constants (*K_d_*) for the *best hits* were calculated from CSPs observed using a ^15^N HSQC titration method. The fragments chosen as the final set of *best hits* were RK104, RK144, RK192, RK207, RK208, RK246, and RK395. Dissociation constants were within expected ranges from high µM to low mM as previously reported by other investigators for this fragment size class [45,50]. RK395 had the highest affinity with 0.51 ± 0.37 mM for PaGRX. 

The ligand efficiency for these fragments varies from 0.22 to 0.34 $\frac{\mathrm{kcal}}{\mathrm{mol}\;\times \;n}$. With the exceptions of RK104 and RK246, dissociation constants and L.E. were found to correlate well with STD NMR data, as shown in Table 1. Among the bacterially selective hits, RK395 exhibited the most promising results against PaGRX and thus became the main focus of this study. RK395 has about four-fold higher affinity for PaGRX over hGRX1, and eleven fold over BrmGRX. The second best hit, RK207, was found to bind with BrmGRX with two and half fold higher affinity than with hGRX1 and about two fold higher than PaGRX [6]. Upon close scrutinization of the rSTD%, *K_d_* and L.E. values for each best hit (Table 1), most of the hits were found to bind more tightly with PaGRX as compared to other two proteins, suggesting that PaGRX is a more druggable target.

### 2.5. NMR-Based Modeling of Target Protein

**Figure 6:**
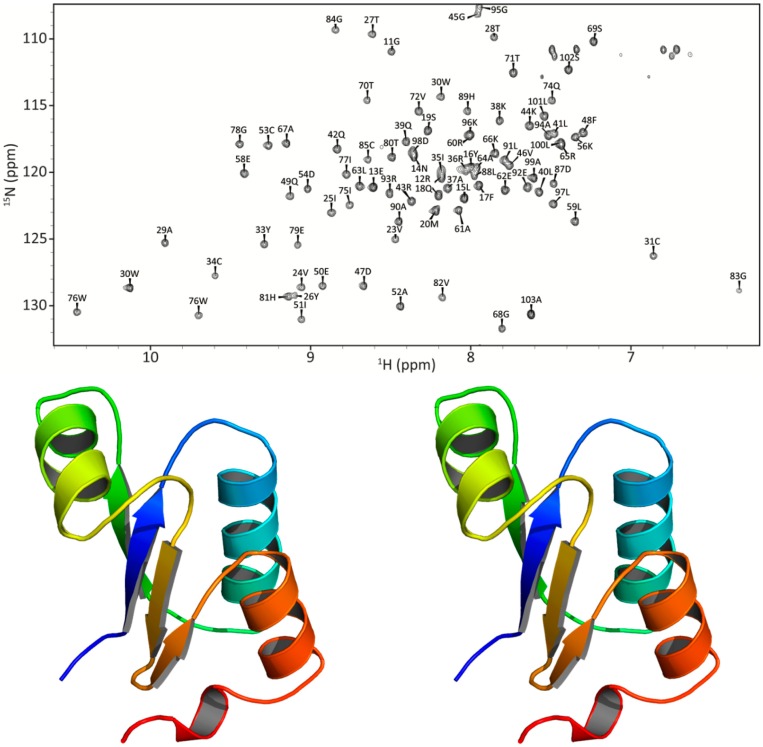
NMR-based model of apo PaGRX in stereoview and its ^15^N HSQC with complete backbone assignments.

**Figure 7:**
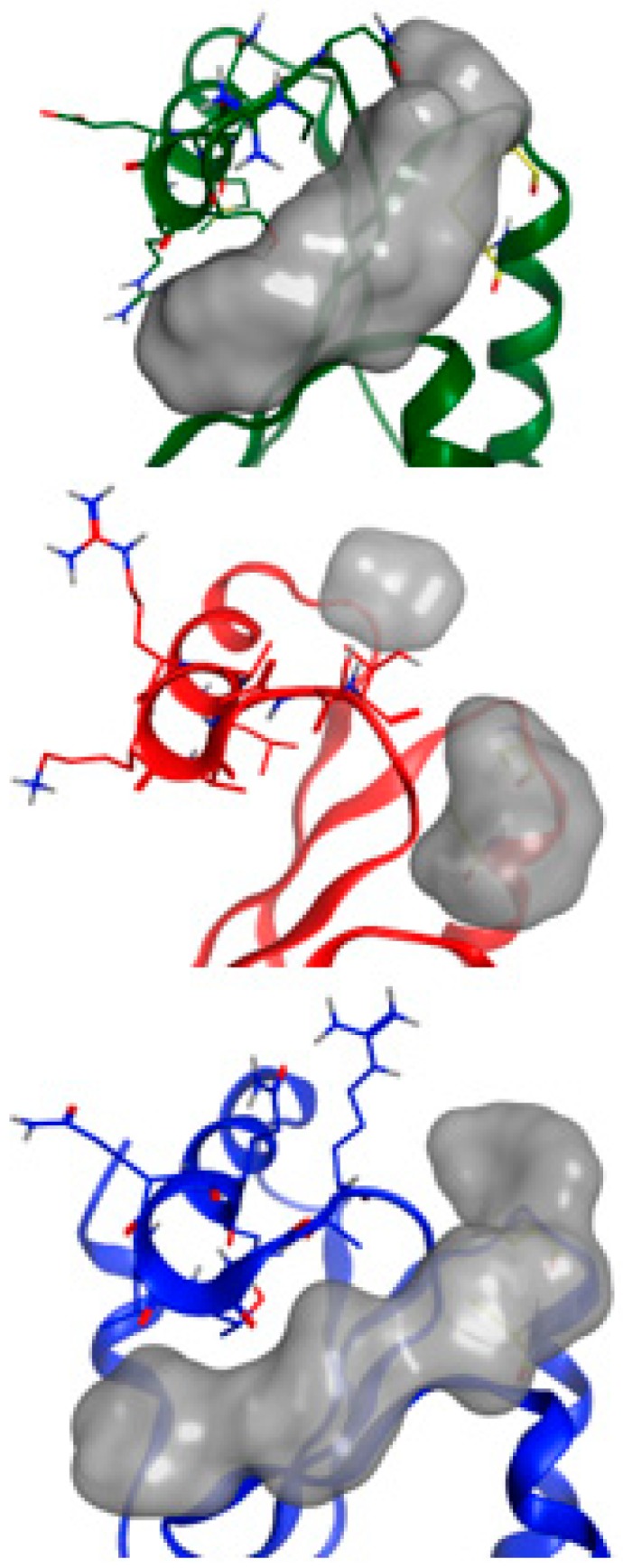
FTMap results for each GRX. Consensus clusters far away from the active site were removed and surface plots were generated over the remaining clusters. Green: BrmGRX, Red: PaGRX, and Blue: Hgrx each have residues from the sequence divergent alpha-2 region rendered as sticks showing their influence or lack thereof over the shape of the active site.

After confirmation of the PaGRX specific hit, a preliminary model of PaGRX’s structure was generated to serve as a template for comparing binding pockets among the orthologous proteins and to aid in prediction of lead fragment binding orientation. For assignment and model building, ^15^N/^13^C labeled samples of PaGRX at −400 µM were produced by recombinant expression in *E. coli*. Triple resonance NMR spectra were collected on a Varian INOVA 750-MHz magnet with triple resonance ^1^H^13^C^15^N cryoprobe. The standard array of triple resonance and NOESY experiments were collected for determining protein structure [51] with distance restraint calibration and assignments optimized by the combined automated NOE assignment and structure determination module (CANDID) of the CYANA software package [52,53]. This structure determination process was initiated during the screening process and fragment hits were compared to this preliminary ensemble before that structure was technically ready for PDB deposition. A full NMR structure determination and refinement for PaGRX is anticipated in the near future and will be presented in a subsequent paper. The PaGRX ensemble used during ligand epitope identification can be considered more like an NMR-validated homology model and further experiments to gain additional restraints for the terminal PaGRX helix are needed before this structure could be deposited in the PDB. Nevertheless, this preliminary NMR-based model compares favorably with prior GRX structures and gives a starting structure to work from in fragment binding epitope identification [4,54,55]. In combination with CSP’s this model is adequate to determine a coarse binding site for RK395 on the protein’s surface and is certainly more accurate than a model derived solely from sequence/structural homology. Figure 6 displays a stereo image of the PaGRX model used to identify fragment binding epitopes and complete backbone assignments for PaGRX in a ^15^N-HSQC. PaGRX adopts the ubiquitous thioredoxin fold: βαβαββαα with a mixed antiparallel and parallel β-sheet flanked by three helices. The CPYC active site residues are in their expected location at the N-terminal end of the first α helix. Both cysteines in the active site have Cα chemical shifts greater than 56 ppm and Cβ shifts less than 32 ppm, indicative of cysteines in a reduced state [56]. Differences in the amino acid sequence define shape and depth of the nearby shallow groove and hence the hot spot architecture identified by FTMap in that region for each protein (Figure 7) [57]. These structural inconsistencies combine to define different binding site shapes and binding energies contributing to ligand selectivity. While the differences are not enough to affect glutathione binding, they can be exploited for FBDD.

### 2.6. Identification of the Binding Pose of the Winning RK395 Fragment with PaGRX

**Figure 8:**
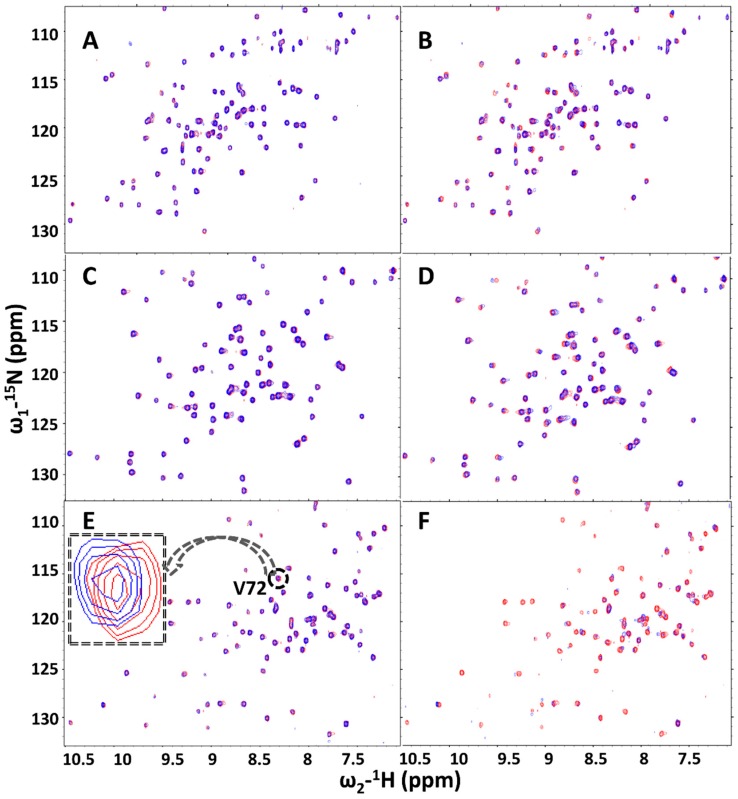
(**A**,**B**) are the ^15^N HSQC spectra for 0.5 mM and 5 mM of fragment RK395 against 0.25 mM ^15^N hGRX1 respectively; (**C**,**D**) with 0.25 mM of ^15^N BrmGRX; and (**E**,**F**) with 0.25mM of PaGRX. Broadening effects observed for many residues of PaGRX, indicating intermediate binding. (Red: Apo, blue: Holo).

**Figure 9:**
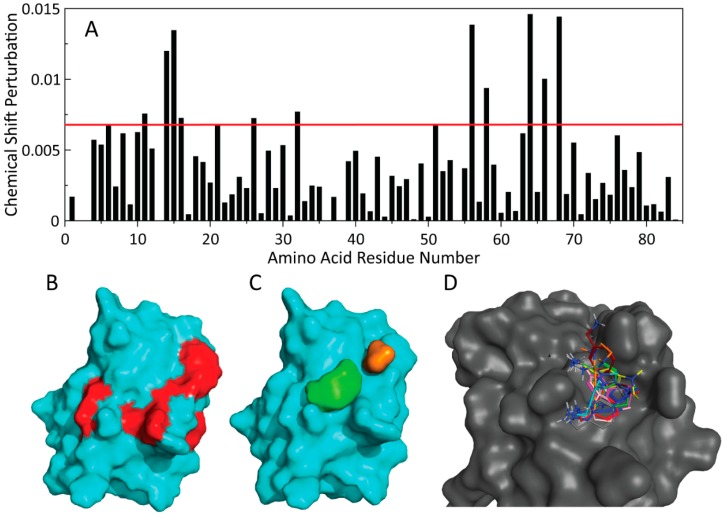
(**A**) Calculated CSP values from 0.2 mM PaGRX bound with 0.5 mM RK395, the red line indicates shifts that are greater than two standard deviations. (**B**) Residues shifted above two standard deviations mapped onto the surface of PaGRX. (**C**) These shifted residues correlate with two hot spots (orange and green) identified by FTMap. (**D**) FTMap clusters were used as a template for initial docking before poses were refined using CSP and STD data.

^15^N-HSQC titration experiments with sub-stoichiometric to excess RK395 concentrations were conducted with PaGRX. With respect to the NMR time scale, RK395 displays intermediate exchange with PaGRX. Obvious broadening effects can be observed for several residues, a phenomena that does not occur with BrmGRX or hGRX1 titrated with RK395 under the same conditions (Figure 8). CSPs and broadening were observed for several peaks, most belonging to residues in or near the active site. When these perturbations are mapped onto the surface of PaGRX, the binding pocket defined is just next to but not directly within the active site (Figure 9A,B). This suggests a binding site proximal to the active site that enables the possibility for chemical elaboration to grow the lead into it.

In our previous FBDD investigation of a GRX we used molecular dynamics (MD) simulations, computational CSP calculations, and fragment docking to predict the binding mode of the species selective lead compound, RK207, to BrMGRX [6]. We were reluctant to use a similar strategy with a preliminary NMR-based model of PaGRX but wanted to determine if other tools such as FTMap, an epitope mapping tool that takes advantage of docking of probe structures, to gain a hint at the binding mode of RK395 with PaGRX. CSPs produced by the titration of RK395 into a solution of PaGRX produced CSPs that correlate with two of the consensus clusters identified by FTMap (Figure 9C). We used these two clusters as restraints in docking simulations of RK395 with our preliminary NMR-based PaGRX model. A sample of potential poses refined into bins of 0.5 Å RMSD of atom positions is shown in Figure 9D. In many of these poses the primary amine of RK395 was oriented toward the active site cysteines of RK395. We considered this portion of the fragment to be a reasonable candidate for chemical extension with an acrylamide warhead group into the active site.

### 2.7. Acrylamide Warhead Development 

There are two parts to an effective cysteine trap type warhead drug: (1) driving group, i.e., the majority of the molecule that confers target selectivity and (2) the electrophile warhead, i.e., an irreversible thiolate reactive moiety. Acrylamides are established as a cysteine trap thiolate reactive group, but their effectiveness against GRXs had not been previously observed. To demonstrate that acrylamides are reactive with GRXs we collected ^15^N-HSQCs after reaction with excess acrylamide to identify which residues experience modifications. A multiday incubation period was allowed to ensure complete reactivity of the relatively slow unmodified acrylamide. Perturbations were observed via ^15^N HSQC and calculated as before, a majority of CSPs were around the active site while the active site cysteine peaks themselves broadened away completely, indicating localized unwinding of the helix in the CPYC active site region and intermediate exchange between multiple conformers on the NMR chemical shift timescale. Spectra were recollected after the sample had been extensively dialyzed to remove free or weakly bound acrylamide. Identical spectra after dialysis demonstrated that the observed adduct was covalent, or at least very long lived and stable, and that the acrylamide reacts with thiolates in the CPYC active site. Since RK395 binds rapidly and non-covalently while acrylamide reacts slowly but covalently, we proposed to link these two together to try to meld their properties and make a comparatively fast reacting covalent adduct to demonstrate the selectivity of RK395 for PaGRX.

**Scheme 1:**
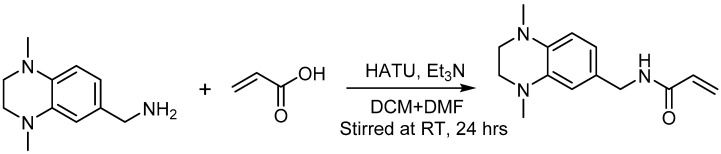
Synthesis scheme for converting RK395 to RK395ACP by extension with acrylic acid.

Using acrylic acid, an acryl functional group was added at RK395′s amide group and the product RK395ACP was produced as shown in Scheme 1. A coupling reaction between α,β-unsaturated carbonyl compound (i.e., acrylic acid) with the amine containing compound (RK395) was performed in the presence of 1-[Bis(dimethylamino)methylene]-1H-1,2,3-triazolo[4,5-b] pyridinium 3-oxide hexafluoro-phosphate (HATU) and Triethylamine (Et_3_N) as a catalyst. The tertiary amine of triethylamine acts as a base, assisting in the formation of oxybenzotriazole (OBT) activated ester with HATU [58]. This OBT activated ester further reacts with RK395 to form a yellowish-brown product (50%) that was purified via flash chromatography, dried, and dissolved into dimethyl sulfoxide (DMSO) for reactions with the GRX proteins. The purity of the final product (Rk395ACP) was checked with high-resolution mass spectrometry (HRMS) and 1D ^1^H NMR and reported in supplementary Figures S4–S5, respectively.

**Figure 10:**
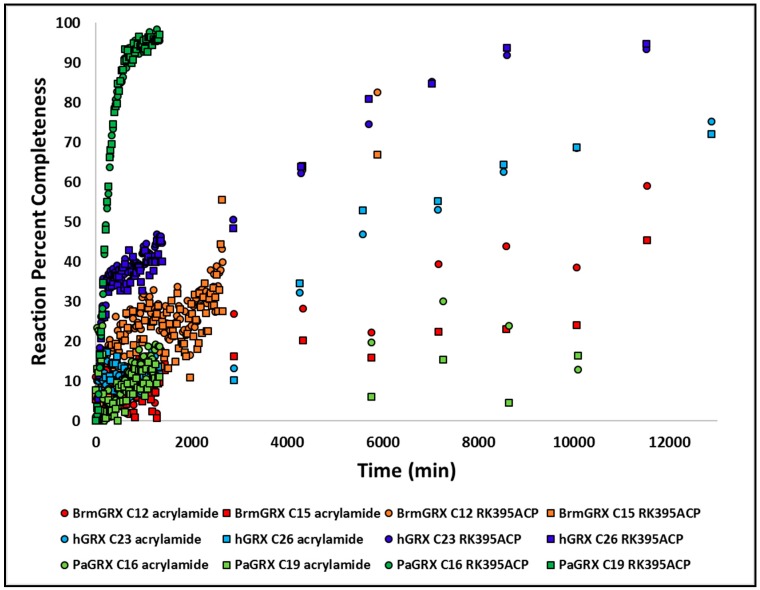
Kinetic study of RK395ACP and acrylamide reacting with the three GRXs. RK395ACP displays significant selective reactivity for PaGRX.

^15^N HSQCs titration experiments were again performed for the fully functional warhead compound (Figure 10). The rate of reaction for this compound was much faster than for naked acrylamide suggesting that the driving group positions the acrylamide moiety into or near the active site to enhance its reaction rate. Again, a cluster of CSPs that flank active site residues are observed as well as broadening of the active site cysteines, suggesting that RK395ACP reacts with PaGRX in the same location and manner as unmodified acrylamide, albeit much faster. To quantitatively determine how much of a rate enhancement the RK395 driving group affords to the mature warhead, kinetic studies for the RK395ACP and acrylamide were observed via HSQC against the three orthologous proteins. In the absence of a driving group, acrylamide reacts very slowly with PaGRX with a reaction half time of about 20 h (Figure 10). In contrast, RK395ACP reacts much more quickly with a 1000-fold improvement in rate, suggesting that the RK395 driving group is either positioning the acrylamide for optimal reaction or increasing the residence time of transiently bound warheads to statistically favor reactivity. RK395ACP also showed faster reaction kinetics for PaGRX over the other two orthologs in a manner that is proportional to the relative *K_d_*’s for the RK395 driving group. Spectra of the PaGRX that had reacted with RK395ACP and then dialyzed to remove excess warhead were unchanged from the pre-dialysis spectra indicating that, like the unmodified acrylamide, the adduct formed between RK395ACP and PaGRX is either covalent of at the very least very long lived. Since the most significant RK395ACP induced broadenings and perturbations are largely confined to the resonance in or near the CPYC active site (Figure S6) we interpreted this as forming an adduct with one or both of the active site cysteines.

### 2.8. Summary of Findings

This work examines the relative binding preferences of a small fragment library against three orthologous GRX proteins and modification of a lead molecule for irreversible inhibition of a bacterial GRX by using an acrylamide warhead. Affinity, selectivity, and binding sites of these fragments were determined by 1D and 2D NMR experiments and MD simulations. By combining acrylamide’s strong tendency to form alkylated cysteine adducts with an FBDD-derived driving group, a relatively rapid process for generating warhead compounds for the selective inhibition of thiolate using enzymes has been developed. The overall hit rate derived from STD-NMR is slightly higher than the hit rate reported by Wielens et al., [42] but smaller than reported by Barelier et al. [45]. This difference could be influenced by the type of target proteins, differences in buffer and/or pH, and differences in physicochemical properties of fragments [42,59]. The experimental methods employed also had variance in hit rates. Ligand-based 1D NMR experiments gave a higher number of hits compared to target-based 2D NMR. This can be attributed to the sensitivity issues between experimental methods as reported by others [50].

Comparing structural properties of the selective and non-selective hits also revealed interesting trends suggesting that scaffold preference may contribute to selectivity. Cyclohexyl benzenes (scaffold XVIII) and cyclohexyl methyl benzenes (scaffold XXV) are preferred non-selective hits (Figure 3). These substructures primarily bind using hydrophobic interactions, resulting in higher logP values for common hits [40]. Most of the selective hits preferred two scaffolds: dicyclo pentadiene (scaffold XX) and phenyl cyclopentadiene (scaffold XVII). The presence of several functional groups independent of scaffold correlate with hit rates as well. A majority of the selective hits found from STD screening contain either one or two of the following functional groups: hydroxyl, carboxyl, and amines. Similarly, hits that were found to show significant CSPs in 2D experiments also contain carboxyl, hydroxyl, or amine groups. This suggests that hydrogen bonding and/or formation of salt bridges play an important role in the interaction between fragment and protein to produce significant CSPs [26]. This is further demonstrated by the lower log P of selective hits, telling that enthalpic contributions to binding may be significant compared with non-selective fragments. Most likely, enthalpic and entropic contributions from multiple functional groups on the lead combine with variations in shape and flexibility between the different scaffold preferences described above to generate these ortholog selectivity trends.

^15^N HSQC experiments have the potential to not only determine binding affinity of fragments, but also for identifying the location of their binding site on target protein by mapping CSPs onto the surface of the protein structure [25,26]. Perturbation data from RK395 titrations with PaGRX showed intermediate exchange on the NMR timescale. Mapping CSPs to the structure suggests a hot spot binding site occupying the shallow grove formed between α_2_ and its flanking loops and the N-termini of α_1_ and α_3_ (indicated by red patches in Figure 9B). A similar region had been previously proposed to be druggable based solely on its structure [4] and was also the major binding site in the other orthologs (Figure S3). It was expected that this hot spot would serve as the same binding epitope used by RK395ACP’s driving group after chemical elaboration with the acryl functional group. 

Fragment selectivity was investigated using three NMR techniques: STD, trNOE and ^15^N HSQC. STD NMR is more sensitive, giving 40 hits that were selective to one or two of the orthologous GRXs. Of these 40 STD selective hits, nine of them were confirmed selectively binding with their respective bacterial proteins by trNOE, but not to hGRX1. Similarly, 28 STD selective hits (70%), when titrated in excess stoichiometric ratios, showed small and large CSPs with their respective proteins. Among these 28 hits, seven were chosen as *best hits* on the basis of their significant CSPs. *Kd* and L.E. values of five out of seven best hits were found to correlate with STD data as shown in Table 1; this correlation suggests STD signatures can generally be representative of relative affinities even when obtained with only a single saturation time. Probable reasons for varying hit rates was the use of different methods and the solvent system [60]. This implies that STD results verified with trNOE and ^15^N HSQC experiments provide enhanced understanding of selectivity and binding of these small fragments with their respective ortholog proteins and suggest an appropriate pipeline for obtaining selective fragment hits from a variety of orthologous protein sets.

In theory, a meta-analysis of screens from different researchers on related protein orthologs could give similar information; yet subtle differences in experimental conditions from screen-to-screen would likely introduce significant false positives and negatives. This error would be expected to corrupt the interpretation of selectivity and hence cause such studies to fail. Our strategy to examine the orthologs together circumvents this by identifying sets of fragments with preference for one or more protein. The results indicate that although the frequency of finding selective fragments against orthologous proteins is rather low, it is still possible to get some selectivity at this initial stage of drug design and convert those small fragments into a more selective fragment as we did with RK395. Our results also imply that libraries of at least several hundred fragments are required to observe selectivity. Lastly, this strategy also enables interrogations of general GRX specificity versus those that are PAINS. 

Chemical warheads of the cysteine trap variety are emerging as options for drug discovery [34]. They have been useful for antineoplastics and tumor drug targets [5], but have not been explored extensively for antimicrobial development. While many different types of cysteine trap moieties are available, the acrylamide functional group was proposed to have favorable properties [37]. In the present study, addition of a driving group derived from a FBDD campaign enhances the rate of acrylamide reactivity with GRXs by over a thousand fold, taking a reaction with a half time of days and shifting it to hours. While the fragment precursor had selectivity for PaGRX over BrmGRX and hGRX, the warhead-attached driving group enhanced the rate of reaction with all three proteins. However, RK395ACP reacted most completely and rapidly with its cognate target, PaGRX. This effect is subtle and suggests that multiple rounds of affinity optimization may be required to further propagate selectivity in mature driving group/warhead chimeric drug compounds. One provocative option is the use of linking selective fragments occupying adjacent hotspots to amplify the selectivity of individual fragments. To do this, one would need high throughput methods for protein-fragment structure determination. Such methods will be critical for exploiting the subtle selectivity obtained by FBDD and translating it into effective driving groups for ortholog selective warhead compounds.

## 3. Experimental

### 3.1. Library Design

A library of 463 ligand fragments was purchased from Maybridge (a subset of the Ro3 library), selected on the basis of expected adherence to the “rules of three”, with a Tanimoto similarity index ≥ 0.68. After studying the structures and predicted physical properties of all the fragments, they were arranged into 79 mixture groups of five to seven fragments so that they could be multiplexed with only minimal resonance overlap in 1D NMR spectra. Stock solutions of 200 mM were prepared by dissolving each of the fragments in d6-DMSO solution (except for a few that were made at 100mM because of their low solubility). Degradation and purity of these 463 fragments were checked by 1D NMR at 25 °C using an Agilent DD2 750 MHz NMR spectrometer (formerly of Santa Clara, CA, USA) at a fragment concentration of 500 µM in D_2_O. These spectra were also used as reference spectra during STD analysis and interpretation.

### 3.2. Protein Expression and Purification

An expression vector for BrmGRX was generously supplied by the Seattle Structural Genomics Center for Infectious Disease (SSGCID, Seattle, WA, USA). The expression vectors for hGRX1 and PaGRX were purchased as codon optimized constructs inserted into the pD434 plasmid (DNA 2.0) with a hexahistidine (6His) tag. All three proteins were prepared by recombinant expression in Escherichia coli strain BL21 (DE3) competent cells in M9 minimal media containing ^15^N ammonium chloride and/or ^13^C glucose (Cambridge Isotope Laboratories, Cambridge, MA, USA), to achieve desired labeling schemes. Protein samples labeled with ^15^N and ^13^C were used for structural studies whereas ^15^N only labeling was used for chemical shift perturbation studies.

Protein expressing cells were grown at 37 °C until an OD_600_ value of 0.500 was achieved. The temperature was then dropped down to 18 °C and 0.5 mM IPTG was added to induce protein expression for 12 or more hours overnight. BrmGRX and hGRX1 producing cultures were lysed using a French pressure cell in a 20 mM Tris-Cl buffer at pH 8 containing 20 mM of imidazole, 200 mM NaCl, and 2 mM dithiothreitol (DTT) as a reductant. PaGRX expressing cultures were lysed in 40 mM sodium phosphate buffer at pH 7 with 200 mM NaCl, 2 mM DTT and 20 mM imidazole. The proteins were then separated from the crude lysate using immobilized metal affinity chromatography (IMAC). Proteins were eluted from the columns with 400 mM imidazole, 200 mM NaCl, 2 mM DTT and 20 mM of Tris-Cl at pH 8. The proteins were then dialyzed into the same lysis buffers described above, but without imidazole. Tobacco Etch Virus (TEV) protease was prepared on site and used to remove the 6His tag from hGRX1 during this dialysis step. HRV 3C protease was used to remove the 6-his tag from BrmGRX. Following cleavage, nickel IMAC was again used to separate the 6His from the protein containing solutions. PaGRX did not have any lysis activity with TEV, so the 6His tag was not removed from any PaGRX samples. Instead of a second IMAC purification step, size exclusion chromatography was used after the first IMAC column [61]. All proteins were then dialyzed against 50 mM sodium phosphate buffer at pH 6.5 with 50 mM NaCl for NMR experiments. For warhead binding experiments, the proteins were pre-reduced with a 2 hr incubation using 0.5 mM TCEP prior to dialysis under inert gas.

### 3.3. Ligand-Based NMR Experiments

All ligand-based NMR spectra (STD and trNOE) were recorded on an Agilent DD2 750 MHz instrument with cryo probe. STD and trNOE samples contained a pool of five to seven fragments for BrmGRX and hGRX1 proteins, and ten to twelve fragments per pool for PaGRX. Pools of fragments were used to multiplex the analysis, reducing NMR time, amount of protein used, and volume of deuterated co-solvents expended. For the three GRXs, STD and trNOE sample contained 10 µM of protein and 0.5 mM of each fragment. The buffer system used for all three proteins was 50mM phosphate with 2mM DTT and less than 4% of d_6_-DMSO in D_2_O. 10% d_8_-glycerol was also used to artificially slow down the proteins’ molecular tumbling rates. These were recorded at 6.2 ^°^C to further enhance this effect in the presence of glycerol. STD screening was performed using a 50 ms Gaussian shape pulse which was used to saturate multiple local frequencies. Prior knowledge about the proton chemical shifts for proteins helped to tune these shaped pulses for the methyl resonance frequency pattern [20]. Experiments were conducted with 64 scans using an acquisition time of 0.682 s and a STD saturation period of 2 s. The off-resonance frequency was applied at −14.24 ppm and on-resonance frequency was applied at −0.74 ppm for all three proteins. Each STD experiment was preceded by 1D NMR experiments as a control to check fragment stability in the sample. For trNOE experiments, relaxation delay of 2.1 s was used with 2048 data points for eight transients. Mixing time of 0.75 s was used with 80 increments and an acquisition time of 0.128 s. ACDlabs (Toronto, ON, Canada) [62] 1D processing software was used to process STD spectra. NMRPipe (Bethesda, MD, USA) [63] and NMRFAM-SPARKY (Madison, WI, USA) [64] were used to analyze trNOE spectra.

Quantitative analysis of STD spectra were performed using relative STD percentage (rSTD%) values, and were calculated as:

(1) $$\mathrm{rSTD}\%=\frac{\left(I_{off}-I_{on}\right)}{I_{off}}\times 100\%$$

here, the intensities of off-resonance and on-resonance peaks are represented as *I_off_* and *I_on_*, respectively. Any fragment that showed rSTD% ≥ 5% in the aromatic region and/or rSTD% ≥ 10% in the aliphatic region were considered hits for that particular protein.

### 3.4. NMR Backbone Resonance Assignments for Three Orthologous Proteins

NMR backbone resonance assignments for BrmGRX (2KHP) and hGRX1 (1JHB) were obtained from the BMRB [65]. PaGRX resonances were assigned using standard protein methodologies. Double- and triple resonance spectra were acquired to obtain sequential connections: ^15^N and ^13^C HSQCs, HNCO, HNCA, HN(CO)CA, CBCA(CO)NH, and HNCACB were first assigned to obtain complete information about backbone atom and α,β carbon chemical shifts. Further side chain carbons and protons were assigned using HCCH-TOCSY, ^15^N and ^13^C NOESY-HSQC, and ^1^H − ^1^H NOESY [66]. All the above experiments were processed and analyzed using NMRPipe and NMRFAM-SPARKY [63,64].

### 3.5. NMR-Based Modeling of PaGRX

NOE cross-peaks were obtained from a simultaneously collected ^13^C and ^15^N NOESY-HSQC in 95% H_2_O:D_2_O (100 ms mixing time) and a two-dimensional ^1^H − ^1^H NOESY in 100% D_2_O (150 ms mixing time). Spectral processing was completed with NMRPipe [63]. NOE assignment and integration were accomplished using NMRFAM-SPARKY [64]. H-bond restraints were derived from retention of amide peaks in the ^15^N-HSQC after protection from exchange with deuterium. Distance restraints were generated in CYANA using volume intensity and upper and lower fraction errors of 0.20 and 0.35, respectively, with lower and upper distance limits of 1.72 and 6.5 Å. Dihedral angle restraints were derived from ^1^Hα, ^15^N, ^13^C, ^13^Cα, and ^13^Cβ assignments using TALOS N [67]. Structure calculation was also performed in CYANA [53]. Out of 100 structures calculated in the final iteration, 20 with the lowest energy were refined in explicit water to the final structure ensemble. The single lowest energy structure was analyzed in MOLPROBITY and PROCHECK [68,69], and visualized with PyMOL [70]. The final ensemble is to be deposited into the PDB [71]. For the purpose of this study the best structure was compared to BrmGRX and hGRX by aligning our best, lowest energy model to the ortholog. While the majority of the structure converged with adequate RMSD and clash scores as determined by MolProbity [72], the terminal helix, which is quite far from the RK395 binding site, still needs some refinement before it would be ready for PDB deposition. Further datasets and structure calculations are required for complete structure determination of PaGRX and will be presented elsewhere.

### 3.6. Protein-Observed NMR Experiments for Fragment Characterization

For each protein, 2D ^15^N HSQC spectra were collected using samples with 0.25 mM of uniformly ^15^N labeled protein in a buffer consisting of 50 mM of phosphate at pH 6.5 and 2 mM DTT in H2O with 5% D_2_O at 25 °C. All spectra were collected using an Agilent DD2 750 MHz NMR spectrometer. The following parameters were used for ^15^N HSQC experiments: relaxation delay of 1s, 8 scans, and acquisition time of 0.0852s, and spectral widths of 12,019.2 Hz in the f2-dimension and 1944.3 Hz in the f1-dimension. Serial titrations of fragments ranged between 0–5 mM depending upon the affinity of fragment toward the proteins. Sample pH was monitored pre and post titration using litmus paper. All 2D NMR experiments were processed and analyzed using NMRPipe and NMRFAM-SPARKY [63,64].

### 3.7. Dissociation Constant (K_d_) Determination

Dissociation constants for seven selective fragments were determined against all three proteins. ^15^N HSQC titration experiments were used to measure CSPs upon ligand-binding. Protein concentration was kept constant at 0.25 mM while fragment titrations were titrated from 0 to 10 mM. The buffers and NMR parameters used were consistent with the ^15^N HSQC CSP experiments described above. Among the ten fragments, six formed complexes with the proteins showing a fast exchange regime relative to the chemical shift timescale. RK395 uniquely showed intermediate exchange with PaGRX, consistent with its modestly higher affinity. The CSPs observed were measured by:

CSP_H_ = δ_H(holo)_ − δ_H(apo)_ (2)

CSP_N_ = δ_N(holo)_ − δ_N(apo)_ (3)

where δ_H(holo)_ and δ_N(holo)_ are the chemical shifts for ^1^H and ^15^N signals in the bound state, respectively. δ_H(apo)_ and δ_N(apo)_ are the chemical shifts for ^1^H and ^15^N signals in ligand-free, respectively [26]. The combined CSP for ^1^H and ^15^N shifts were calculated using the following equation:

(4) $$CSP_{\left(H+N\right)}=\sqrt{\frac{{\left(CSP_{H}\right)}^{2}+{\frac{\left(CSP_{N}\right)}{25}}^{2}}{2}}$$

Combined CSPs were then fit using the non-linear regression equation shown below. Igor Pro 6.36 [73] was used to fit the data and a plot of $CSP_{\left(H+N\right)}$ against [*L_t_*]/[*P_t_*] was constructed.

(5) $$CSP_{\left(H+N\right)}=CSP_{max}\frac{P_{t}+L_{t}+K_{d}-\sqrt{{\left(P_{t}+L_{t}+K_{d}\right)}^{2}-4L_{t}P_{t}}}{2P_{t}}$$

*CSP_max_* represents the maximum CSP that occurred during titration for each fit. *P_t_* and *L_t_* are the total concentration of protein and ligand, respectively [60,74]. *K_d_* values were determined from residue signals that were best fitted with standard deviations below a threshold value of 25% and averaged to measure a final *K_d_*.

### 3.8. Calculating Ligand Efficiency 

Ligand efficiency plays a crucial role in selecting lead fragments from hits to guide further chemical elaboration and development of new drug molecules [75,76]. Ligand efficiency for the fragments was calculated as:

(6) $$LE\;=\;\frac{\Delta G}{N}$$

ΔG is Gibbs’ free energy and is equal to −RTlnK_d_. R is the gas constant 8.314 J/mole × K, T is the temperature in Kelvin, and N is the number of heavy atoms present in the fragment [77,78].

### 3.9. Kinetic Studies of Warhead Reactivity 

Kinetic binding studies with acrylamide and RK395ACP against all three GRXs were performed using ^15^N HSQC. After collecting an apo reference spectrum, the ligand was titrated into solution and 52 sequential 30 min experiments were immediately collected for each sample. If after that point the reactions were determined to be incomplete additional spectra were collected individually in regular intervals as instrument availability permitted. Each sample contained a 1:1 stoichiometric concentration of lead compound to protein in an aqueous buffer containing 40mM of phosphate buffer at pH 7.0 and 8% D2O. As described above the proteins were put into a reduced state prior to ligand titration. All 2D NMR experiments were recorded on an Agilent DD2 750 MHz spectrometer with a cryoprobe at 25 °C and kinetic studies were repeated to check reproducibility. The percent completion of the reaction as monitored by loss of signal in the active site was normalized for aggregation using Equation (7):

(7) $$\%\;Completeness=100\times \left[\frac{\left({\frac{\mathrm{Cys}}{\mathrm{Gly}}}^{\max}-{\frac{\mathrm{Cys}}{\mathrm{Gly}}}^{t_{x}}\right)}{{\frac{\mathrm{Cys}}{\mathrm{Gly}}}^{\max}}\right]$$

where $\frac{\mathrm{Cys}}{\mathrm{Gly}}=\frac{I_{{\mathrm{Cys}}_{t_{x}}}}{I_{{\mathrm{Gly}}_{t_{x}}}}$. In this equation ${\frac{\mathrm{Cys}}{\mathrm{Gly}}}^{\max}$ is the maximum peak intensity ratio of an individual active site cysteine relative to a hydrophobic core glycine over all time points. ${\frac{\mathrm{Cys}}{\mathrm{Gly}}}^{t_{x}}$ is the same ratio taken at time point *x*. The peak intensity ratio is calculated as $\frac{I_{{\mathrm{Cys}}_{t_{x}}}}{I_{{\mathrm{Gly}}_{t_{x}}}}$ where the numerator and denominator are the intensity of the active site cysteine being measured and the intensity of the hydrophobic core glycine respectively.

### 3.10. Molecular Docking

All docking experiments were conducted within Molecular Operating Environment. First, hot spots were predicted using the FTMap web server using the apo structure output from CYANA. Consensus clusters (CC) were then extracted and superimposed onto the original apo structure. CCs that were not within reasonable proximity to residues identified by CSP analysis with RK395 were removed, leaving the CCs defining a large binding pocket partially occupied by GSH under physiological conditions [79]. Docking was conducted with the organic solvent molecules of the CCs serving to define the binding pocket, partially filling the role of a crystallographic ligand used in most site-directed docking simulations. A stochastic conformational library of the conjugate base form of RK395 was generated and used to sample initial binding poses [80]. 20,000 initial poses were sampled using triangle matcher placement, which is exhaustive for small molecules [81]. Poses were ranked using the London dG scoring function and duplicates were removed [82]. Remaining poses were refined using a rigid receptor protocol and rescored using MOE’s GBVI/WSA dG function before final duplicate removal [81].

## 4. Conclusions

This study presented the binding interaction of 463 fragment molecules against three orthologous glutaredoxin proteins, using three different NMR techniques and developing a warhead-based lead by picking the most promising fragment for bacterial protein. Two thirds of the hits obtained from STD NMR, were found as common binders, indicating their versatile nature. Hit rates positively correlate with an increase in size and number of the binding pockets present in the proteins. Our results showed that mM to μM selectivity can be achieved for a few small fragments against orthologous proteins and this selectivity can be amplified by optimizing the promising fragment with appropriate warhead compound. RK395 that binds to PaGRX with moderate specificity, coupled with acrylic acid showed high specificity toward PaGRX, which can be further developed into more potential ROS-enhancing thiol-transferase inhibitory drug candidates against Pseudomonas aeruginosa. Additionally, the favorable enhancement in kinetic reactivity afforded by addition of the fragment-based driving group suggests that even weakly reactive, and hence less inherently toxic, warheads may be viable in such lead molecules. Future studies will investigate optimizing the driving groups further to try to push the kinetics to even faster timescales for the cognate target while maintaining slow reactivity with off-target orthologs.

## Figures and Tables

**Table 1:** Dissociation constants and ligand efficiency for *best hits*.

Structure	Fragment	Selectivity	BrmGRX *K_d_* ± S.D. (mM)	hGRX1 *K_d_* ± S.D. (mM)	PaGRX *K_d_* ± S.D. (mM)	BrmGRX L.E.	hGRX1 L.E.	PaGRX L.E.
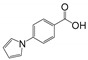	RK207	BrmGRX	0.98 ± 0.24	2.45 ± 0.52	1.90 ± 0.34	0.29	0.25	0.27
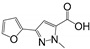	RK144	BrmGRX, PaGRX	5.24 ± 0.92	5.42 ± 0.9	1.92 ± 0.42	0.22	0.22	0.26
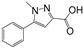	RK246	BrmGRX, PaGRX	10.63 ± 5.18	5.22 ± 1.16	8.48 ± 2.06	0.18	0.21	0.19
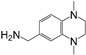	RK395	PaGRX	5.49 ± 3.22	1.86 ± 1.15	0.51 ± 0.37	0.22	0.27	0.32
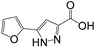	RK104	hGRX1	0.90 ± 0.15	2.89 ± 0.47	0.72 ± 0.16	0.32	0.27	0.33
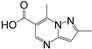	RK208	hGRX1, PaGRX	2.28 ± 0.30	1.61 ± 0.19	0.56 ± 0.08	0.26	0.27	0.32
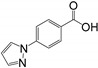	RK192	hGRX1, PaGRX	5.56 ± 0.51	5.05 ± 1.27	5.26 ± 1.12	0.22	0.22	0.22

## Supplementary Materials

Supplementary materials can be accessed at: https://www.mdpi.com/1420-3049/25/1/147/s1.

## Abbreviations

FBDD	fragment-based drug discovery
STD	saturation transfer difference
rSTD%	relative saturation transfer difference
NMR	nuclear magnetic resonance
HSQC	heteronuclear single quantum correlation
SAR	structure activity relationship
BrmGRX	*Brucella melitensis* glutaredoxin
hGRX1	human glutaredoxin 1
PaGRX	Pseudomonas aeruginosa glutaredoxin
HTS	high throughput screening
logP	log of partition-coefficient
*K_d_*	dissociation constant
L.E.	ligand efficiency
CSPs	chemical shift perturbations
trNOE	transferred nuclear overhauser effect
