# Supplementary material for: Identifying Ortholog Selective Fragment Molecules for Bacterial Glutaredoxins by NMR and Affinity Enhancement by Modification with an Acrylamide Warhead
Source: Molecules. 2019 Dec 30;25(1):147. doi: 10.3390/molecules25010147 (PMC6983068; doi:10.3390/molecules25010147)
Supplement: Supplementary file 1 [file molecules-25-00147-s001.pdf]

Supporting information

Ram B. Khattri[1,†], Daniel L. Morris [2,†,ǂ], Stephanie M. Bilinovich [3], Erendra Manandhar [4], Kahlilah R. Napper [2], Jacob W. Sweet [2], David A. Modarelli [2] and Thomas C. Leeper [5,*]

[1] Department of Physiology and Functional genomics, University of Florida, Gainesville, FL 32610, USA; rbk11@ufl.edu

[2] Department of Chemistry and Biochemistry, The University of Akron, Akron, OH 44325, USA; danmorris4127@gmail.com (D.L.M.); krn14@zips.uakron.edu (K.R.N.); jws81@zips.uakron.edu (J.W.S.); dmodarelli@uakron.edu (D.A.M.)

[3] Department of Pediatrics and Human Development, Michigan State University, East Lansing, MI 48824, USA; bilinovi@msu.edu

[4] Department of Chemistry, Berea College, Berea, KY 40404, USA; emanandhar@gmail.com

[5] Department of Chemistry and Biochemistry, Kennesaw State University, GA 30144, USA

* Correspondence: tleeper@kennesaw.edu; Tel.: +1-470-578-2258; Fax.: +1-470-578-9137

† These authors contributed equally to this work.

ǂ Current Address: Biochemistry and Biophysics Center, National Heart Lung and Blood Institute, National Institutes of Health, Bethesda, MD 20814, USA.

Table S1. Residues perturbed by 20-fold excess of fragments with all three proteins.

Fragments	Residues Perturbed for BrmGRX	Residues Perturbed for hGRX1	Residues Perturbed for PaGRX
RK207[a]	R51, F57, G69, S70, D71, D72, L73, A75, L76, E77	E4, F5, N7, S8, Y25, C26, R28, A29, N52, E56, I57, Y60, Q62, T65, G66, A67, R68, T69, V73, K77, G81, G82, D85, L95, L99	V23, T28, A29, Y33, C34, I35, Q39, C53, D54, L63, L66, S69, T70, T71, V72, Z74, I75, W76, G84, C85, D87, L88, H89, A90, L91, E92
RK144[a]	A22, A38, R51, S52, F57, G69, S70, D71, D72, L73, A75, L76, E77	E4, F5, S8, Q11, C26, R28, A29, N52, E56, I57, Y60, Q62, G66, A67, R68, T69, V73, K77, I80, G81, G82, D85, L95, L99	V23, T28, Y33, C34, I35, Q39, C53, D54, L63, L66, S69, T70, T71, V72, Z74, I75, G84, C85, D87, L88, H89, A90, L91, E92
RK246[a]	R13, R51, F57, G69, S70, D71, D72, A75	E4, F5, N7, S8, C26, R28, A29, N52, I57, Y60, G66, R68, T69, V73, K77, G81, G82, D85, L95, L99	V23, T28, A29, Y33, C34, I35, Q39, C53, D54, L63, L66, S69, T70, T71, V72, Z74, I75, G84, C85, D87, L88, H89, A90, L91, E92
RK395[a]	R13, Y18, C19, E32, R51, F57, G69, S70, D71, D72, L73, A75, L76, E77	E4, S8, Y25, C26, R28, A29, N52, E56, Y60, T65, G66, R68, T69, V73, K77, G81, G82, D85, L95	V23, T28, A29, C31, Y33, C34, I35, Q39, C53, D54, L63, L66, S69, T70, T71, V72, Z74, I75, W76, T80, H81, V82, G83, G84, C85, D87, L88, H89, A90, E92
RK104[a]	I10, T12,R13, Y18, C19, A22, L26, E32, A38, R51, S52, F57, Q59, I62, G69, S70, D71, D72, L73, A75, L76, E77	E4, F5, S8, Q11, Y25, C26, R28, A29, V46, N52, E56, I57, Y60, Q62, T65, G66, A67, R68, T69, V73, K77, I80, G81, G82, D85, S92, E94,L95, L99	V23, T28, A29, C31, Y33, C34, I35, Q39, C53, D54, L63, L66, S69, T70, T71, V72, Z74, I75, W76, T80, H81, G84, C85, D87, L88, H89, A90, L91, E92
RK208[a]	I10, T12,R13, Y18, C19, A22, L26, E32, A38, R51, S52, F57, Q59, I62, G69, S70, D71, D72, L73, A75, L76, E77	E4, F5, S8, Q11, Y25, C26, R28, A29, V46, N52, E56, I57, Y60, Q62, T65, G66, A67, R68, T69, V73, K77, I80, G81, G82, D85, S92, E94,L95, L99, I102	V23, T28, A29, C31, Y33, C34, I35, Q39, C53, D54, L63, L66, S69, T70, T71, V72, Z74, I75, W76, T80, H81, G84, C85, D87, L88, H89, A90, L91, E92
RK192[a]	R13, L26, A38, R51, F57, G69, S70, D71, D72, L73, A75, L76, E77	E4, F5, S8, Y25, C26, R28, A29, N52, E56, I57, Q62, T65, G66, A67, R68, T69, V73, K77, G81, G82, D85, L95, L99	V23, T28, C31, Y33, C53, D54, L63, L66, T71, V72, Z74, I75, G84, C85, D87, L88, H89, L91, E92

[a] Blue letters indicates residues showing large CSPs or broadening effects.

.

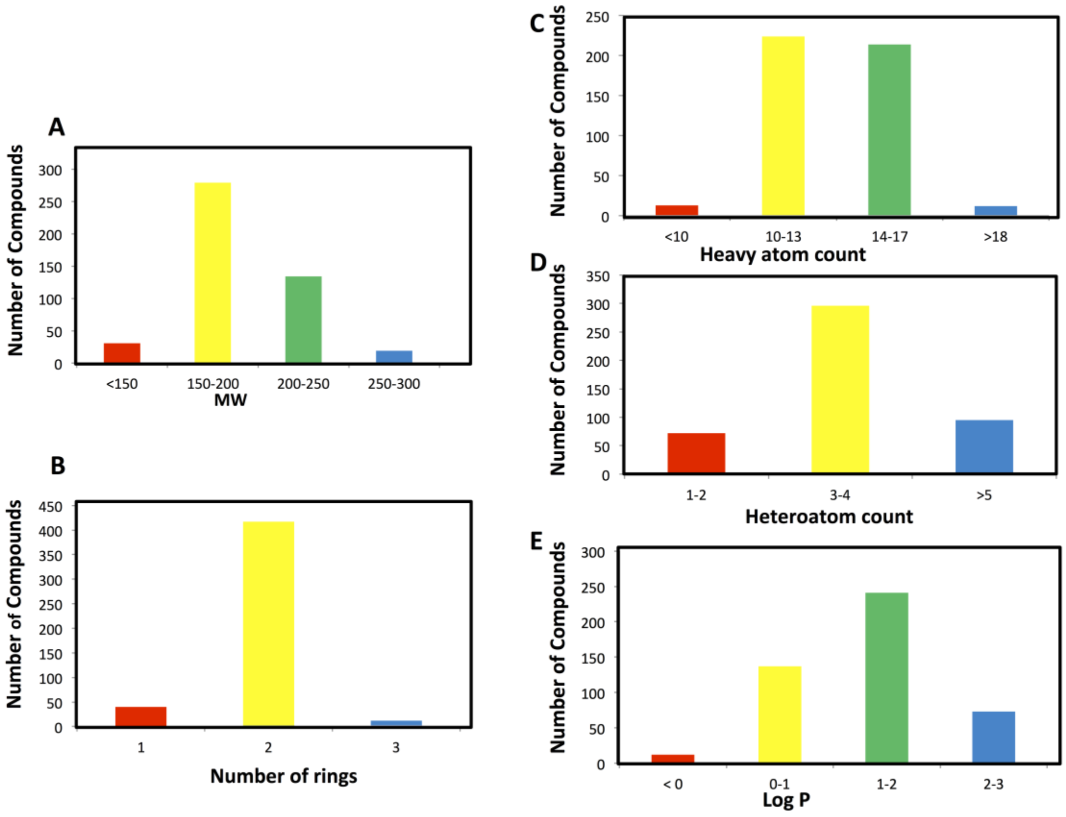

Figure S1. Diversity of different parameters in the library. Diversity of: (A) molecular weight, B) number of rings C) heavy atom count D) heteroatom count and E) partition coefficient (logP) values.

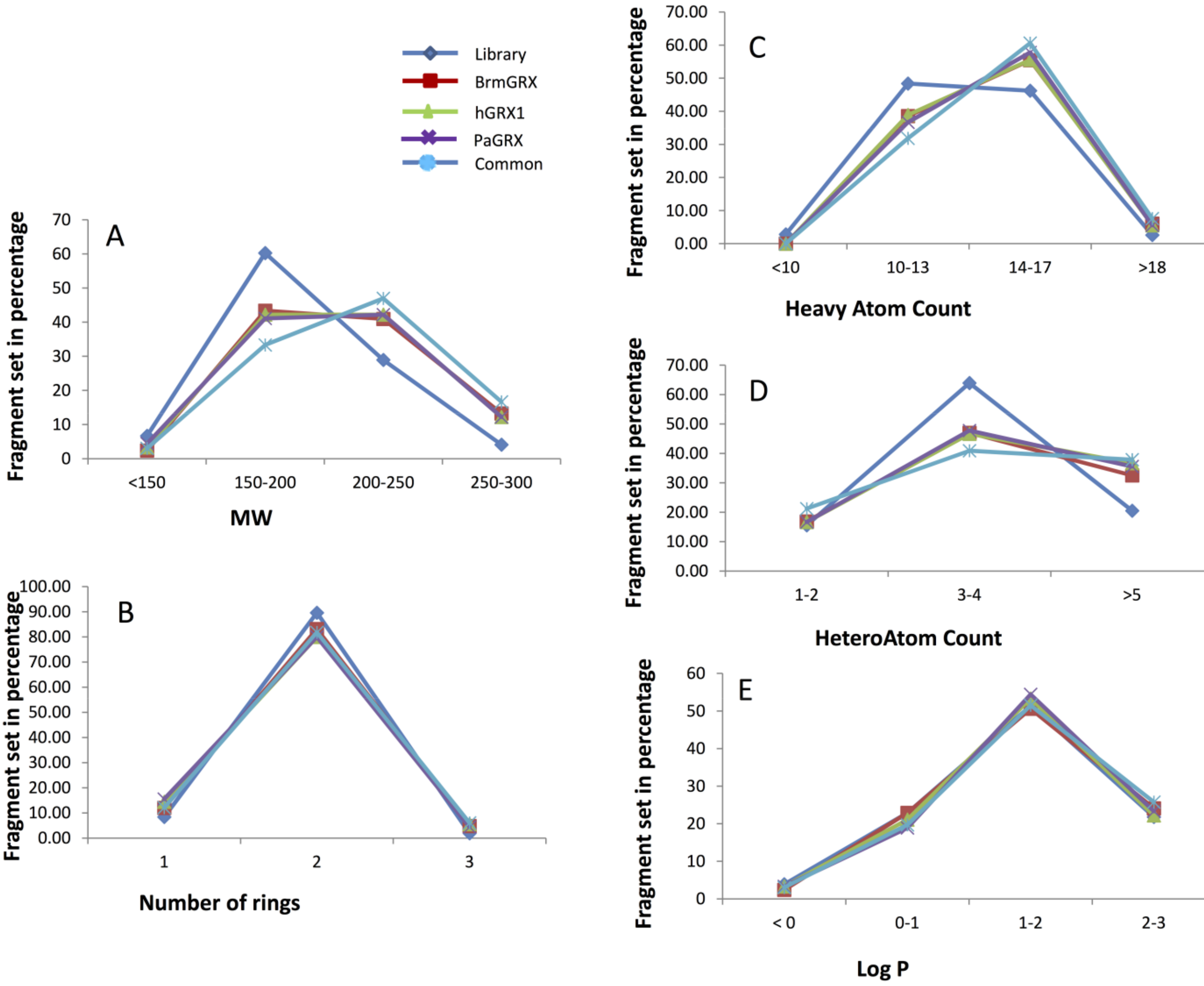

Figure S2. Diversity of average physicochemical parameters in-between hits and library: (**A**) molecular weight, (**B**) number of rings (**C**) heavy atom count (**D**) heteroatom count (**E**) logP; for hits of three orthologous proteins, common hits and library.

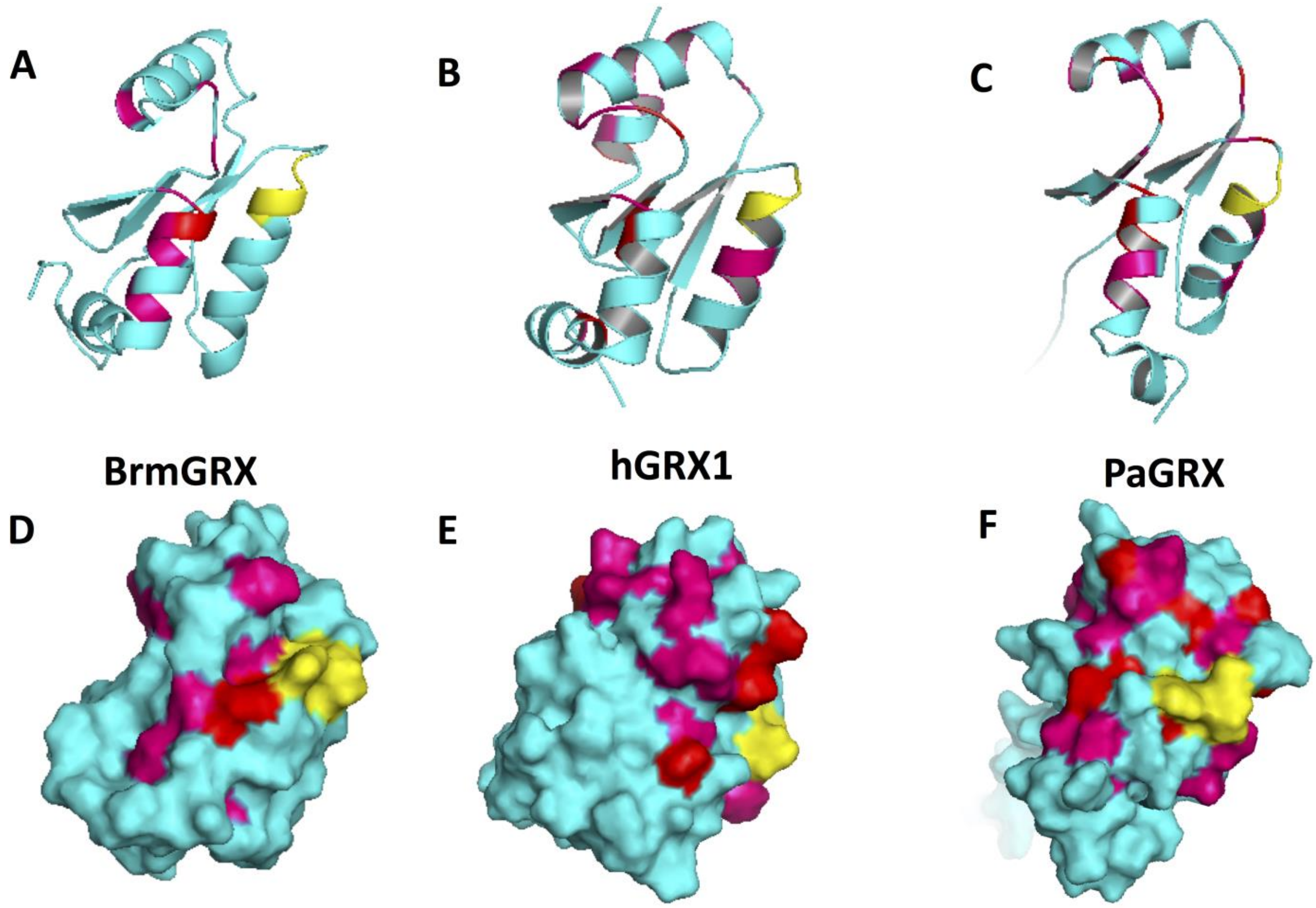

Figure S3. 2D NMR CSPs mapping. (**A**), (**B**), and (**C**) represents secondary structures; and (**D**), (**E**), and (**F**) are the surface structures with CSPs mapping for BrmGRX, hGRX1, and PaGRX, respectively. Yellow patch: conserved active site; red patches: residues with CSPs ≥ 0.1 ppm; and pink patches: residues with CSPs ≤ 0.1 ppm with large excess of fragment.

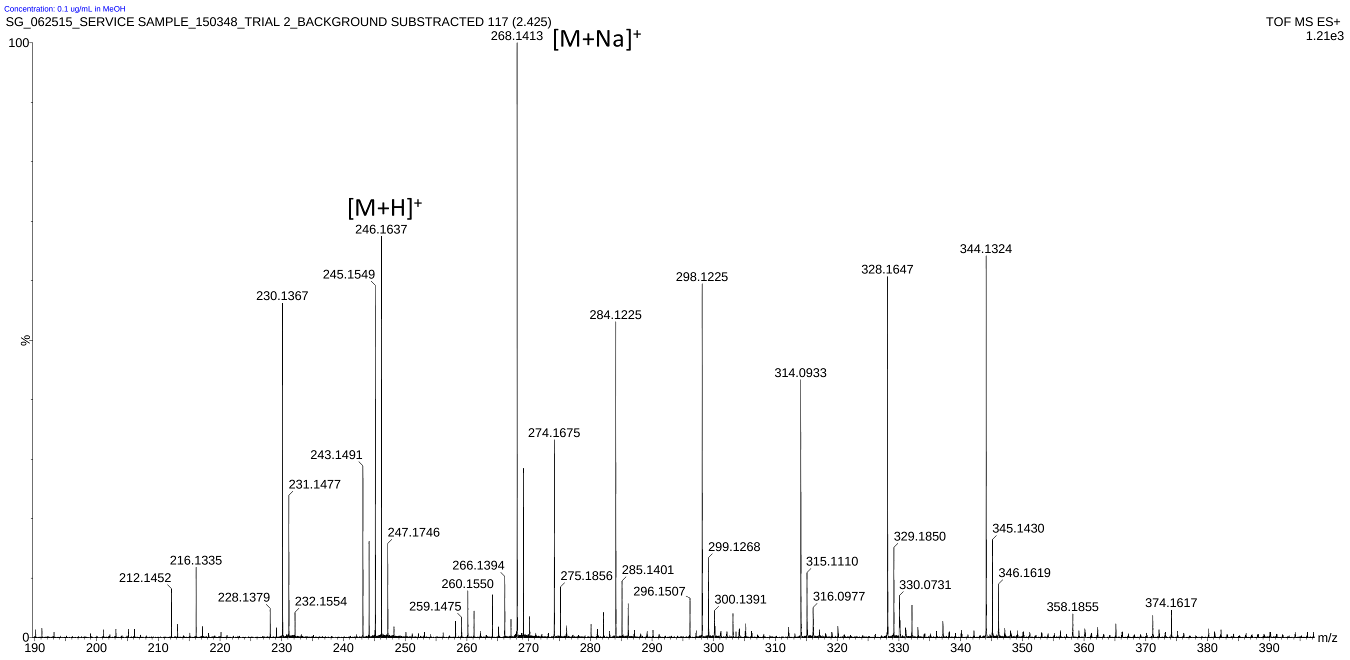

Figure S4. HRMS for RK395ACP in methanol. HRMS calculated for $C_{14}H_{19}N_3O$ 246.1606, found 246.1637 ($M + H^+$).

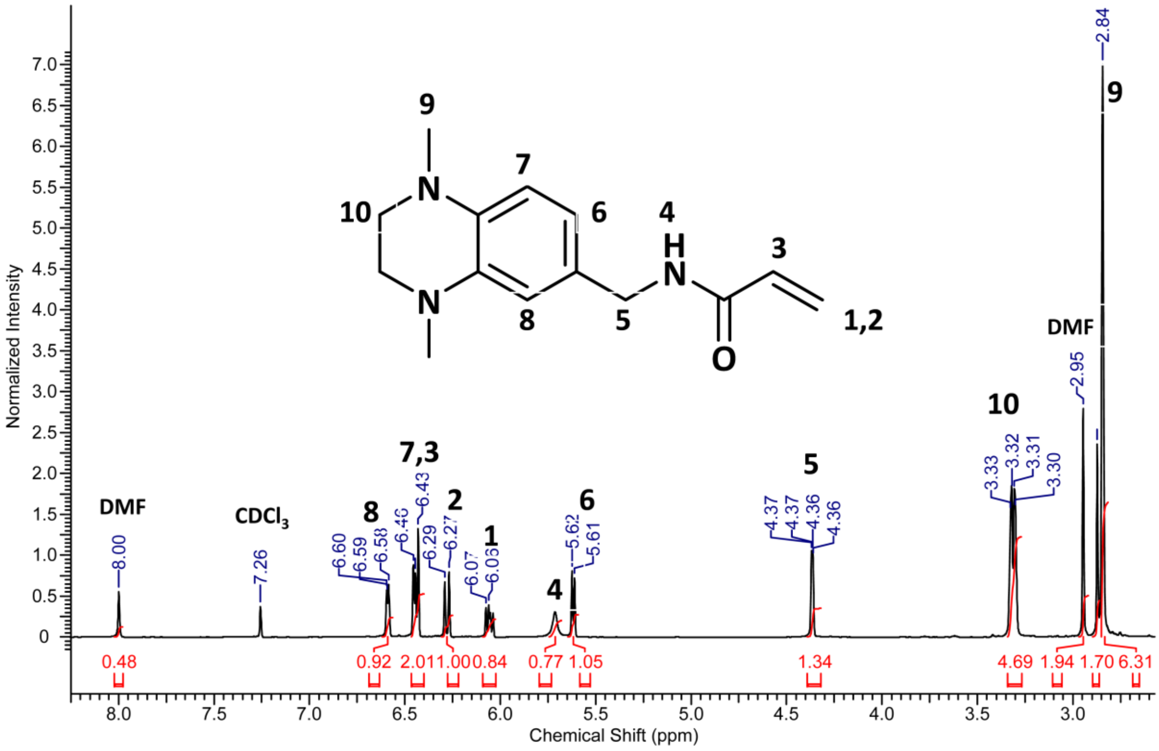

Figure 5. 1D ^1H NMR for RK395ACP.

^1H NMR (750 MHz, $CdCl_3$) δ 6.59-6.60 (d, 1H), 6.45-6.46 (d, 1H), 6.43 (s, 1H), 6.27-6.29 (d, 1H), 6.03-6.07 (m, 1H), 5.65 (s, 1H), 5.61-5.63 (d, 1H), 4.37 (s, 2H), 3.31-3.32 (d, 4H), 2.84 (s, 6H)

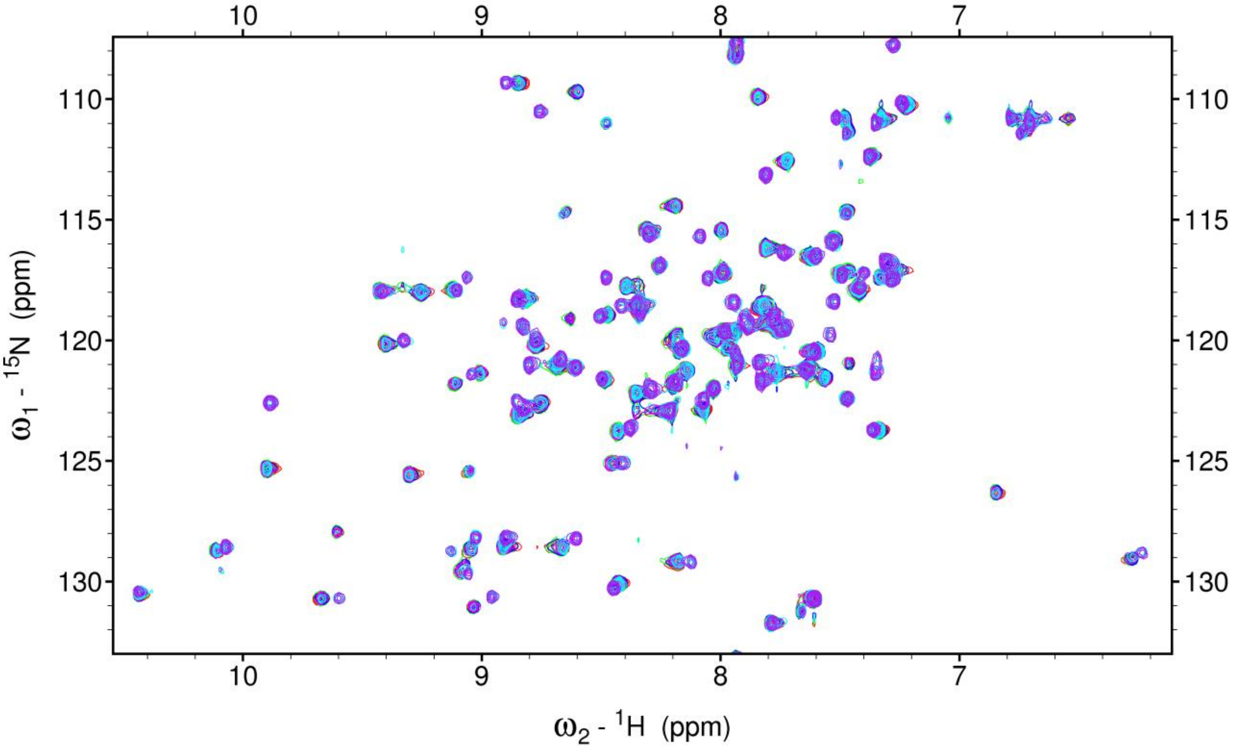

Figure S6. Spectral overlays for PaGRX reacted with RK395 ACP—Green: 0 h, blue 1 h, magenta: 2 hours, cyan: 6 h, purple: 24 h.
